# Recent advances in human papillomavirus vaccines and therapeutic strategies: Combating cervical and non-cervical cancers

**DOI:** 10.1016/j.gendis.2025.101880

**Published:** 2025-10-17

**Authors:** Md Rezaul Islam, Abdur Rauf, Most Nazmin Aktar, Md Naeem Hossain Fakir, Sadiya Islam Trisha, Asraful Islam Asif, Md Harun Or Rashid, Md Ibrahim Khalil Al-Imran, Gazi Kaifeara Thufa, Farhana Prodhan Emu, Hassan A. Hemeg, Hanan A. Ogaly, Rekha Thiruvengadam, Seung-Hyun Kim, Muthu Thiruvengadam

**Affiliations:** a Department of Pharmacy, Faculty of Health and Life Sciences, Daffodil International University, Daffodil Smart City, Birulia, Savar, Dhaka 1216, Bangladesh; b Department of Chemistry, University of Swabi, Anbar 23561, Khyber Pakhtunkhwa, Pakistan; c School of Natural Sciences, Macquarie University, Sydney, New South Wales, Australia; d Department of Clinical Laboratory Sciences, College of Applied Medical Sciences, Taibah University, Al-Medinah, Al-Monawara 41477, Saudi Arabia; e Chemistry Department, College of Science, King Khalid University, Abha 61421, Saudi Arabia; f Centre for Global Health Research-Helix Research Lab, Department of Neonatology, Saveetha Medical College and Hospital, Saveetha Institute of Medical and Technical Sciences (SIMATS), Thandalam, Chennai, Tamil Nadu 602105, India; g Department of Crop Science, College of Sanghuh Life Science, Konkuk University, Seoul 05029, South Korea; h School of Engineering, Macquarie University, Sydney, New South Wales, Australia

**Keywords:** Cervical cancer, Gene editing, Human papillomavirus, Immunotherapy, Prophylactic vaccines, Therapeutic vaccines

## Abstract

Human papillomaviruses (HPV) are a major cause of several cancers, particularly cervical cancer, and remain a serious public health challenge, particularly in low-resource countries. In addition to cervical cancer, HPV is linked to vulvar, vaginal, penile, anal, and oropharyngeal cancers, especially in men. The integration of HPV into the human genome plays a key role in cancer development. This review highlights the progress in HPV vaccination and new treatment approaches for non-cervical HPV-related cancers. Current vaccines provide strong protection against cervical cancer, and next-generation vaccines aim to protect against more types of cancer-causing HPV. New immunotherapy strategies, such as DNA-based vaccines and antigen-specific immunotherapy, are being developed to more effectively target HPV-driven cancers. Promising methods, such as CRISPR/Cas9 gene editing, therapeutic vaccines, and immune checkpoint inhibitors, have shown success in early research and clinical trials. Among these, DNA vaccines stand out as cost-effective and scalable solutions for treating HPV-related tumors. This review also explores the biology of HPV-related cancers, global trends, and the latest advances in prevention and treatment. To reduce the burden of HPV-related diseases, a combined approach involving vaccination, early detection, and personalized treatment is essential. Ongoing research on therapeutic vaccines, gene therapies, and immune-based treatments could greatly improve the management of HPV-related cancers, potentially lowering their global impact. Expanding these innovations in clinical practice may significantly reduce the global burden of HPV-related malignancies.

## Introduction

**Figure 1:**
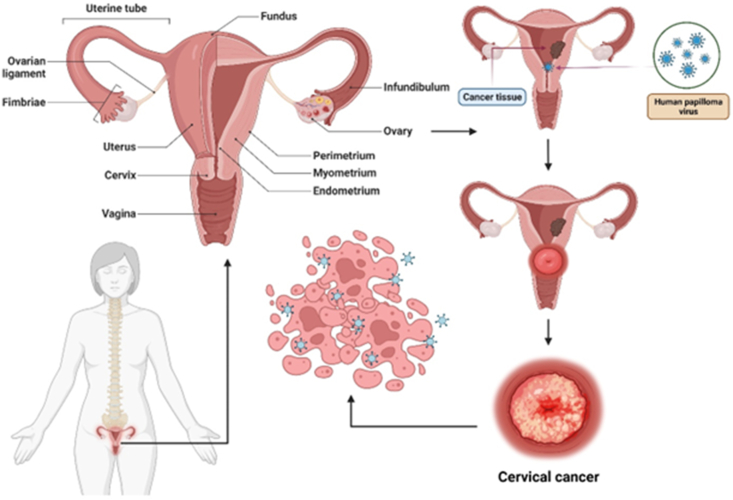
Representation of cervical cancer.

Cancer is a leading cause of morbidity and mortality worldwide, with a growing burden in both high- and low-income countries.^1^ According to GLOBOCAN 2022, the global cancer burden has reached an estimated 20 million new cases and 9.7 million deaths, with human papillomavirus (HPV)-related cancers accounting for a substantial proportion, particularly cervical and oropharyngeal cancers.^1^^,^^2^ Approximately 15%–20% of all cancers are attributed to viral infections, with HPV being one of the most prominent oncogenic viruses.^3^ Persistent infection with high-risk HPV, particularly HPV16 and HPV18, is a necessary factor for several epithelial cancers, including cervical, anal, vulvar, penile, and oropharyngeal cancers.^4^ HPV is a non-enveloped, double-stranded DNA virus that belongs to the papillomaviridae family.^5^ It infects basal keratinocytes in the epithelium, typically through microabrasions.^4^ The life cycle of a virus is closely linked to epithelial cell differentiation.^6^ In oncogenic HPV types, viral proteins E6 and E7 interfere with the host tumor suppressors p53 and pRb, respectively, disrupting cell cycle control and promoting genomic instability.^7^ A critical incident in HPV-induced carcinogenesis is the integration of viral DNA into the host genome, leading to sustained expression of E6 and E7, which influences malignant transformation.^8^ This integration also disrupts viral regulatory regions, further enhancing oncogene expression and contributing to oncogenesis. Although cervical cancer remains the most common HPV-associated malignancy globally, non-cervical cancers, particularly oropharyngeal squamous cell carcinoma (OPSCC), have emerged as a major concern, especially in high-income countries.^9^ HPV-positive OPSCC is currently the most common HPV-related cancer in men in many regions, including North America and parts of Europe.^10^ For instance, the United States has seen a marked increase in HPV-positive OPSCC cases over the past two decades, surpassing the incidence of cervical cancer.^9^^,^^11^ European data suggest that HPV16/18-related oropharyngeal cancers account for more than 15,000 new cases annually in men.^12^ Despite the curability of early-stage cervical cancer with surgery, chemotherapy, or radiotherapy, treatment success for recurrent or metastatic disease remains limited.^13^ Similarly, HPV-positive head and neck cancers are often diagnosed at advanced stages, where prognosis is poorer and treatment is more complex.^14^ HPV infection significantly influences the development of numerous epithelial cancers, such as cervical carcinoma (Fig. 1). Prophylactic vaccines, such as the nonvalent HPV vaccine, have significantly reduced the incidence of HPV infections and precancerous lesions.^15^ However, prophylactic vaccination is typically administered before sexual intercourse, and although coverage has improved, its uptake remains suboptimal in many regions.^16^ Moreover, due to the long latency between HPV infection and cancer development, vaccinated cohorts may take decades to exhibit measurable reductions in cancer incidence.^17^ Therapeutic HPV vaccines aim to stimulate cell-mediated immunity, particularly cytotoxic T lymphocyte responses, against HPV oncoproteins E6 and E7, which are constitutively expressed in HPV-transformed cells but are absent in healthy tissues.^18^ Several vaccine platforms, including DNA-, RNA-, peptide-, and viral vector-based formulations, are under preclinical and clinical investigation.^19^ Recent studies have also explored host factors contributing to HPV pathogenesis, such as genetic susceptibility, immune evasion mechanisms, and major histocompatibility complex (MHC) polymorphisms.^20^ Altered expression of ion channels and dysregulation of cellular pathways, such as PI3K/Akt and Wnt/β-catenin, have been implicated in HPV-mediated oncogenesis.^21^ This review highlights the current knowledge of HPV infection, associated malignancies, and the emerging therapeutic landscape, with an emphasis on therapeutic vaccination strategies aimed at overcoming the limitations of current HPV cancer treatments.

## Human papillomavirus

### Basic characteristics

**Figure 2:**
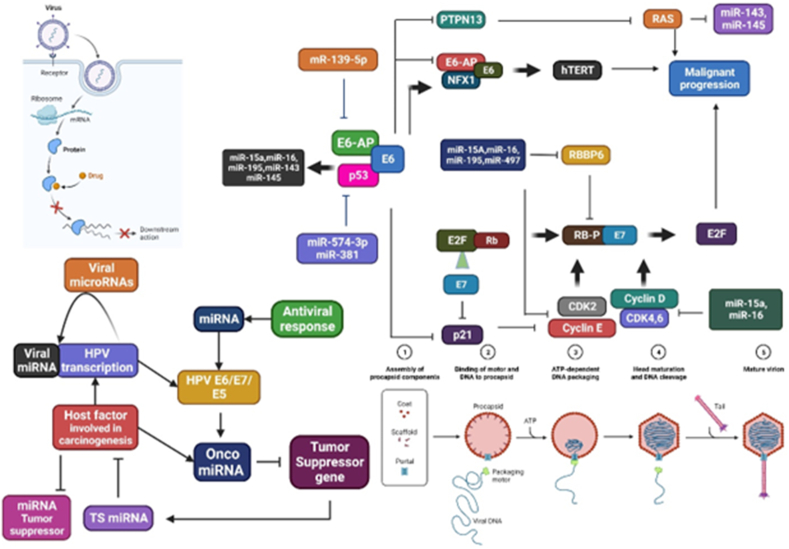
Potential miRNA-mediated regulatory mechanism is depicted schematically that might be active during the development of cancer caused by HPV.

Tiny deoxyribonucleic acid (DNA) viruses, known as HPV, primarily infect the epithelial tissues of a wide variety of animal species, including humans.^22^, ^23^, ^24^ The absence of tissue culture systems for the proliferation of HPV has inhibited research on these viruses. The human wart virus is the only HPV responsible for wart development, and local environmental factors influence variations in wart shape and clinical progression.^25^ HPV genomes consist of eight genes for replication proteins, transcription (Fig. 2), transformation-related proteins, double-stranded circular DNA (8 kb), and a non-coding regulatory long control region.^26^ Papilloma is caused by HPV, a double-stranded circular DNA virus. HPV contains icosahedral capsids with nonstructural protein-producing genes for assembly, release, transcription, and replication. L1 and L2 genes produce viral cellular proteins, and the virus consists of 72 pentameric capsomeres. L2 protein plays a lesser role in virion production.^27^^,^^28^ Viruses accumulate in the cell nuclei, leading to malignant transformation, cancer initiation, and synthesis. The E6 and E7 proteins are involved in viral DNA insertion into the host genome.^29^ HPV-induced carcinogenesis is primarily triggered by two oncoproteins, E6 and E7, which are encoded by HPVs.^30^ HPV oncoproteins E5, E6, and E7 disrupt telomere preservation, cell cycle regulation, DNA damage, and genomic disorders, leading to the development of cancer. They also inhibit tumor suppressor pathways and induce apoptosis. Cytokines alter oncoproteins E6 and E7, thereby affecting cell growth and immune response. HPV viral proteins and integration promote chromosomal abnormalities and cellular immortalization.^31^^,^^32^ Figure 2 schematically depicts the potential miRNA-mediated regulatory mechanisms that may be active during HPV-induced cancer development.

### The cycle of the virus

**Figure 3:**
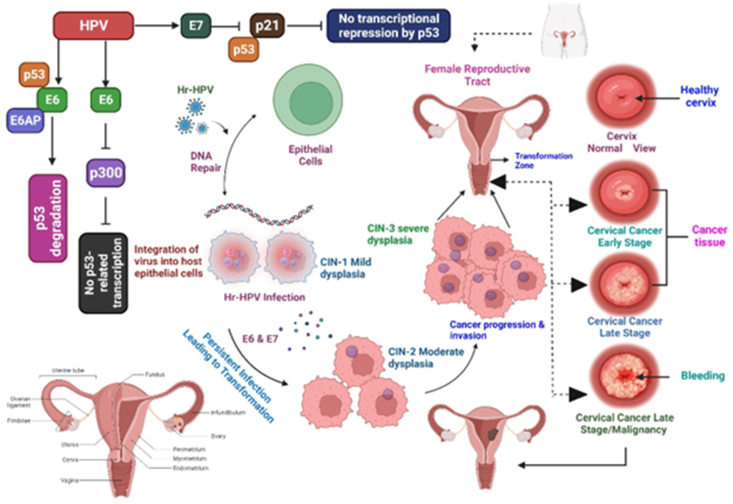
Cervical cancer and its stages.

The majority of cervical cancer cases were caused by only a few HPV types. HPV-16 and HPV-18 are HPV strains that are extremely potent carcinogens.^33^, ^34^, ^35^, ^36^, ^37^ The final differentiation of keratinocytes is necessary for HPV to complete its life cycle. Basal keratinocytes are infected by the red pentagon virus, which initiates their life cycle after penetrating the basal lamina. Basal cells contain the viral genome, an extra-chromosomal replicon before cell development, and early HPV protein expression increases. Differentiating cells are induced to enter the cell cycle again, and the viral genome is improved to a high copy number.^38^ A cutaneous or mucosal injury allows HPV to enter the body and spread from person to person or from mucosa to mucosa.^27^ When it interacts with integrin 6, a cell surface receptor found in epithelial stem cells and basal cells, it can spread the infection among these cells. Infected cells still contain the majority of the HPV genome and precancerous tumors at the episomal stage.^39^ Keratinocytes in the skin are susceptible to HPV infection, which can spread through mucous membranes and other body tissues. HPV infection targets keratinocytes, which suppress the immune system and allow them to remain dormant.^27^ The virus may remain dormant in squamous cells even after the immune system clears, necessitating immunosuppression for contagiousness and differentiation of the infected host's basal epithelial cells.^27^ HPV infections are usually temporary and asymptomatic, and the immune system eventually removes them. Non-oncogenic HPV infections last for eight months, whereas oncogenic ones have a median duration of 13 months. HPVs can be divided into cutaneous and mucosal types, with mucosal forms causing cervical neoplasia and anogenital warts.^40^ Figure 3 illustrates the various stages of cervical cancer.

### Mucosal tumors associated with HPV

**Figure 4:**
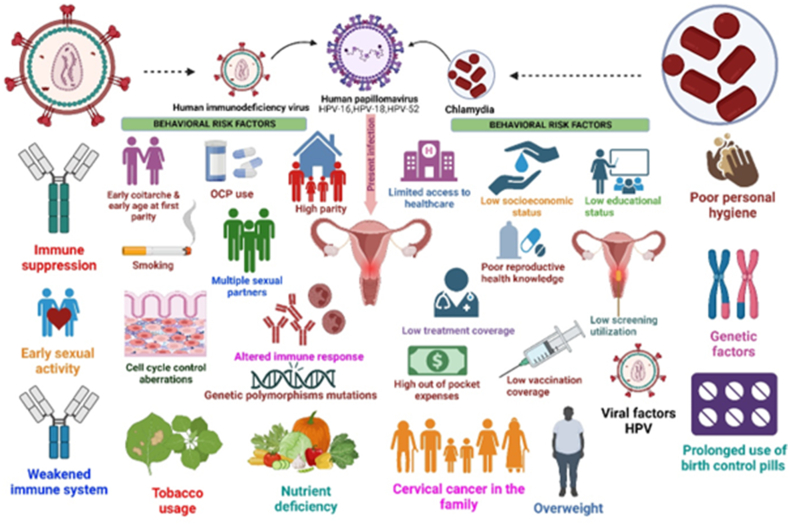
Risk factors for cervical cancer and high-risk HPV infection.

HPV infection in this genus causes anal tumors due to squamous intraepithelial lesions affecting almost all of the cervix. Some penile, vulvar, and vaginal cancers have been linked to the HPV genus. HPV-16, the most prevalent oncogenic subtype linked to these diseases, is the most prevalent form of HPV and has the highest risk of cancer development.^41^ A study of anogenital cancers in North America, Europe, Asia, and South America revealed that 75% of HPV-positive non-cervical anogenital cancers were HPV 16-positive. Young squamous cells in the cervix and anus are particularly susceptible to HPV oncogenic strain infection. In the transformation zone, the non-keratinizing squamous epithelium of the anus changes into columnar epithelium.^27^ Oncogenic HPV can infect the female vaginal canal without a cervical transformation zone, thus affecting both hysterectomy-induced and non-hysterectomy-induced women. HPV can spread to other locations and is linked to oral squamous cell carcinoma in people under 50.^41^ Malignancy may be indicated by malignant tumors in which HPV enters the host genome. HPV integrates consist of bounded and nonrecurrent regions of amplification. HPV-positive malignancies exhibit genomic instability and are strongly linked to HPV insertional breakpoints, which are characterized by chromosomal translocations, deletions, inversions, and intrachromosomal rearrangements. HPV significantly contributes to cancer maintenance; however, it is not sufficient to convert epithelial cells independently. HPV infection is crucial for the genetic and epigenetic processes that occur after cancer genesis and cellular change.^32^ Anal cancer and nearly persistent viral infections with cancer-causing HPV strains are the primary causes of cervical cancer in every case.^39^ High-grade dysplasia and cancer can be caused by various factors, including persistence, oncogenesis, age above 30 years, multiple HPV strain-related diseases, immunosuppression, and cigarette use.^29^ HPV-related penile and vulvar tumors develop earlier in life, have a basaloid pathology, are unaffected by p53 mutations, and are associated with sexual risk factors. The study found a link between high-grade squamous intraepithelial lesions, cervical cancer, and oral HPV infections in women.^41^ Figure 4 shows the risk factors for high-risk HPV infections and cervical cancer.

### HPV infection

The mechanism of HPV infection is intriguing and unusual. New research has revealed that the unique characteristics of the process are adaptations to viral lifestyle traits, such as limiting productive life cycles to terminally differentiated stratified squamous epithelia. Early studies utilized non-viral particles (virus-like particles), which can be formed by overexpressing the L1 primary capsid protein.^42^ The viral life cycle begins when basal epithelial cells are infected and is influenced by their differentiation.^43^ The vaginal tract can be infected with approximately 40 different HPV types.^44^ HPV-associated cervical infections (Table 1) typically go undetected because of the host's cell-mediated immunity, disappearing a year or two after the onset.^45^ A woman with high-risk HPV types but normal cytology has a lower 18-month clearance rate for HPV-16 infections than other high-risk HPV types.^46^ HPV prevalence among females with normal cytology is higher in less developed regions (15.5%), with HPV-16 and HPV-18 infections primarily occurring in these regions.^47^

**Table 1:** An overview of recent research evaluating the presence of HPV-associated viral and pathogenic co-infections in various types of cancer.

HPV co-infection with additional pathogens	Techniques for detecting	Number of patients	Incidence of cancer	Clinical consequence	Finding	Reference
HIV/HPV co-infection	Prospective cohort study	300	Grade 2 cervical intraepithelial neoplasia	HPV persistence 46%	Evaluated the 1-year outcomes of cervical cancer screening and treatment in HIV-positive women using primary high-risk HPV testing	195
Co-infection of HPV with HSV and (*Chlamydia trachomatis, Trichomonas vaginalis*)	PCR	300	At least 3% different co-infections	Cervical cancer	Identified HPV infections and co-infections with other significant sexually transmitted infections among women in northern Peru's public health system	196
Co-infection of HIV-1, HSV-1/-2, and six other pathogenic sexually transmitted infections	Hybrid Capture-2 and multiplex PCR STD direct flow chip assays	205	At least two pathogens co-infected (52.7%).	Cervical cancer	Raised public awareness about the severe impact of sexually transmitted infections on women's health through early detection, effective treatment strategies, and prevention	197
HPV co-infection with *Chlamydia trachomatis*, *Neisseria gonorrhoeae*, *etc*.	PCR assays	44	HPV co-infection with *Chlamydia trachomatis*, *Neisseria gonorrhoeae*, *etc*.	Cervical cancer	Demonstrated the prevalence of HPV infection and co-infection with other bacterial infections among Filipino cervical cancer patients	198
*Mycoplasma hominis* and *Ureaplasma urealyticum* HPV co-infection	Molecular analyses	120	HPV (83.9%). The co-infection of *Ureaplasma urealyticum* and HPV is greater in metastatic cancer.	Cervical cancer and intraepithelial neoplasia	Determined the correlation between co-infection of *Mycoplasma hominis*, *Ureaplasma urealyticum*, and HPVs in women and cervical lesions	199
HPV/EBV co-infection	Sequencing and type-specific PCR/nested-PCR	166	Head and neck squamous cell carcinoma	Co-infection occurred in two cases	Evaluated the prevalence of HPV and EBV co-infection in oral and oropharyngeal squamous cell carcinomas	200

### Burden of HPV

Infection-related malignancies account for 16.6% of the global cancer cases, with 3.3% in developed countries and 32.7% in developing countries. HPV-based cervical cancer screening has revolutionized the diagnosis, treatment, prevention, and prophylactic vaccination of cervical neoplasia.^48^ HPV is the leading cause of cervical cancer, with 93% of biopsy samples from over 900 consecutive cases containing HPV DNA.^49^ A study found that only 7% of models tested negative for HPV DNA, indicating a low prevalence of HPV-free cervical malignancies.^50^ HPV is linked to vaginal and anogenital cancers, with high-grade vaginal intraepithelial neoplasia (VAIN-3) lesions present in 64%–91% of cases. HPV-related vulvar and penile cancers cause 60%–90% of cases in young people, whereas less than 10% affect older adults.^44^ Approximately 0.5% of all male malignancies worldwide are pyelonephritis, although 40%–50% of cases are HPV DNA-positive.^51^ HPV has been linked to nononcogenic malignancies. HPV DNA has been detected in head and neck squamous cell carcinoma (HNSCC). HPV-16 was the primary cause of HPV-positive squamous cell carcinomas in the laryngeal (69.2%), oral (68.2%), and oropharyngeal (86.7%) regions.^52^ Ultraviolet exposure, immunosuppression, and HPV strains, especially HPV-5 and HPV-8, are potential contributors to the development of non-melanoma skin cancer.^44^

## Infection-related risk factors

### Usage of oral contraceptives

Understanding the role of reproductive traits and oral contraceptive usage in cervical carcinogenesis requires a deeper understanding of their potential impact on HPV infection onset and persistence. The connection between oral contraceptive use and sexual activity complicates the determination of whether oral contraceptive use causes HPV infections. The study found a weak correlation between HPV-16, -18, and −31 seropositivity and prior oral contraceptive use after adjusting for age and lifetime partner number.^53^ After adjusting for variables, including the number of sex partners, some studies have reported a link between the two.^54^^,^^55^ A study found heterogeneity in the link between combined oral contraceptive consumption and widespread HPV infection.^56^

### The number of sexual partners

A continuous risk factor for HPV infection is an increased number of sex partners. Numerous studies of both women^53^^,^^57^, ^58^, ^59^ and men^60^, ^61^, ^62^ have discovered significant associations between the lifetime number of sex partners and the development of genital HPV. A woman's self-reported HPV infection was positively correlated with her male partner's predicted lifetime sex partners.^57^ The shorter time between meeting a new partner and engaging in sexual activity significantly increased the likelihood of HPV infection in women.^63^ A study indicated that a male partner's age over 1.5 years increases the likelihood of HPV DNA detection in teenage girls and women.^58^ The risk of HPV infection is increased due to behavioral hazards, such as age at first sexual contact, number of partners, and partners' sexual behavior.^64^^,^^65^ Studies suggest that a higher prevalence of high-risk HPV infection and women with multiple infections may be linked to sexual behavior, social status, high parity, lack of barrier contraceptive protection, and prolonged oral contraceptive use.^66^ Co-factors are frequently present and have the potential to influence HPV acquisition, persistence, development, and progression into neoplastic lesions.^67^

### Smoking

Cigarette smoking increases the incidence of cervical neoplasia. A study involving 23 individuals found that current cigarette smokers had a significantly higher risk of developing carcinoma *in situ* and cervical cancer than did non-smokers. The risk of oncogenic HPV type increased with daily smoking frequency and persisted in analyses that focused on women who tested positive for HPV. Smoking duration did not significantly impact the risk of cervical neoplasia, which increased with a decrease in smoking initiation age.^68^ A study found that women who had never smoked had a significantly lower frequency of HPV infection than those who had more sexual partners.^69^ Another study investigating the association between smoking and oncogenic HPV infection revealed no correlation between the number of cigarettes smoked daily and the presence of HPV DNA.^70^

### Male features

HPV infection affects both males and females. Interpretation of studies on HPV infection frequency in men is inhibited by non-standardized collection techniques and diverse patient populations.^71^ A study found that women with multiple male partners had a higher risk of contracting HPV, with unclear prior relationships and an increased risk of incidental infection.^97^ Female respondents with multiple male sexual partners were three times more likely to test positive for HPV than those with monogamous sexual partners.^57^ The combined IARC HPV prevalence surveys found a less significant association, with women in extramarital relationships having nearly 1.5 times higher odds of being HPV-positive.^72^ Older male partners are more likely to be HPV carriers, increasing a woman's risk because of the age gap between her and her first partner.^73^ Men who have sex with men are highly likely to be infected with HPV.^74^, ^75^, ^76^ The incidence of anal cancer among these men is expected to be 44 times higher than that in the general population owing to the high prevalence of anal HPV.^77^

## Potential genetic changes in cancers related to HPV

### Head and neck squamous cell carcinoma

In 2022, the US reported 66,470 new cases of HNSCC, with 15,050 expected deaths due to the disease.^78^ A study in younger American populations (ages 20–44) found that squamous cell carcinomas are more prevalent in other oral cancers than pharyngeal locations.^79^ HPV-driven patients have less genomic complexity than HPV-negative cases, which is linked to significant alcohol and tobacco use.^80^ The poorer prognosis of cancers linked to smoking, p53 mutations, and immunological adjustments may be due to increased genomic complexity. 3q26/28, a transcription-related gene in the squamous lineage, exhibits recurrent localized amplification in both HPV-positive and HPV-negative HNSCC tumors.^81^^,^^82^ Changes in nuclear factor kappa B (NF-κB) transcription factors, which are crucial for angiogenesis, cell migration, and inflammation, are influenced by these changes. Renin angiotensin system mutations are associated with poor prognosis in various cancers.^83^ A total of 17.6% of HPV-positive tumors have fibroblast growth factor receptor 2 (FGFR2) and FGFR3 mutations, including the N569D, N569K, and S249C variants. These mutations are sensitive to FGFR inhibitors.^84^^,^^85^ Receptor-associated factor 3 (TRAF3) has been linked to deletions and truncating mutations in HPV-related HNSCC.^81^ Both innate and adaptive antiviral responses are mediated by TRAF3. Nasopharyngeal carcinomas and hematologic malignancies are linked to uncontrolled NF-κB signaling, which is facilitated by the loss of TRAF3.^86^^,^^87^ Human leukocyte antigen-A (HLA-A) and HLA-B immune response genes are also altered in HNSCC. Additionally, alterations in gene expression have been observed in HPV-positive HNSCC, including RAD51B, ATM, FANCG, FANCA, and FANCD2. The chemo- and radiosensitivity of HPV-positive cancers has been linked to changes in DNA repair genes.^82^

### Cervical cancer

**Figure 5:**
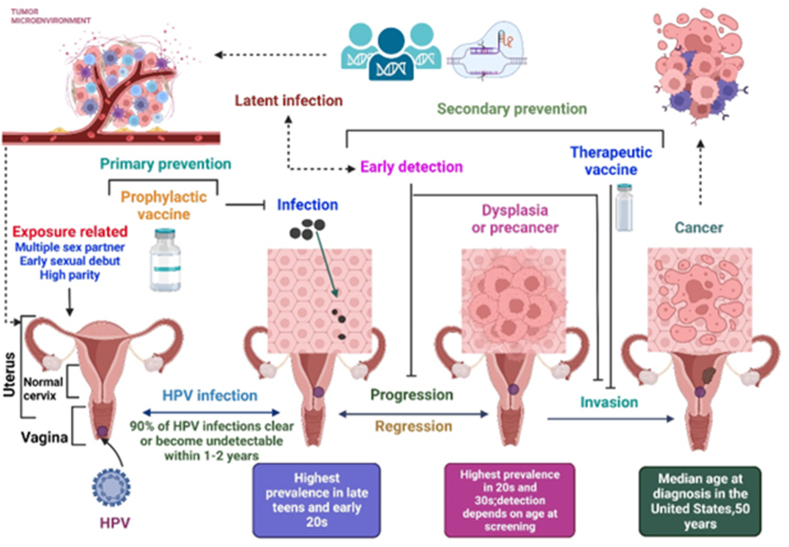
The development and infection of cervical cancer caused by HPV.

Cervical cancer is the fourth most prevalent type of cancer in women.^88^ High-risk HPV strains are the primary cause of cervical cancer, and effective prevention programs include HPV screening and immunization.^89^ Mutations in immune response genes and antigen presentation genes, similar to those found in HNSCC, suggest a potential interaction between HPV infection and squamous cell carcinoma etiology.^90^ Most patients with cervical cancer experience a prolonged asymptomatic period before the clinical manifestation of the disease. Routine screening can detect early cytological changes, potentially preventing the progression of pre-invasive diseases into invasive ones. Physicians can make informed decisions about patients requiring ongoing screening by identifying women at risk of invasive cervical cancer. Patients with sexually transmitted diseases, HPV infection, poor socioeconomic status, multiple sexual partners, and immunosuppression are at higher risk of developing cellular abnormalities.^91^ Figure 5 illustrates the development of cervical cancer due to HPV infection.

## Modern therapies and treatments

### Surgery

The incidence of oropharyngeal cancer, primarily linked to HPV, is increasing, despite the prevalence of head and neck cancer. At least 50% of these cases are caused by HPV.^92^, ^93^, ^94^ The Surveillance, Epidemiology, and End Results (SEER) program predicted a high prevalence of HPV-associated OPSCC, with an estimated prevalence of 71%.^95^ People with HPV-associated OPSCC, who are younger, nonsmokers, and have a high likelihood of long-term survival, may be the most suitable group for minimally invasive transoral surgery.^96^^,^^97^ Replacement of surgical resection as a safe treatment option emphasizes the importance of obtaining surgical specimens for pathological staging and adjuvant therapy selection. The two main transoral surgical procedures used to treat head and neck cancer are transoral laser microsurgery and transoral robotic surgery.^96^, ^97^, ^98^ Transoral laser microsurgery is a potential treatment option for early head and neck cancer.^98^ The method utilizes standard surgical equipment in hospitals, including a CO_2_ laser, operating microscope, and laryngoscope.^98^^,^^99^ The ECOG 3311 clinical experiment evaluated the de-intensification of postoperative radiotherapy after surgical removal of OPSCC caused by HPV.^97^^,^^100^^,^^101^ New transoral surgical methods have been developed to reduce cosmetic deformity, improve function, and enhance the quality of life.^97^^,^^102^ Younger patients are particularly susceptible to long-term treatment issues and need to choose the best treatment option. Radiation-based treatments provide consistent oncologic outcomes; however, their intrinsic morbidities include mucositis, dysphagia, neutropenia, nephrotoxicity, neurotoxicity, ototoxicity, xerostomia, fibrosis, trismus, and osteoradionecrosis.^103^^,^^104^

### Chemotherapy

Only a small percentage of HPV-associated oropharyngeal cancers have persistent or recurrent disease and respond well to standard therapies.^105^ Younger patients often experience post-treatment side effects, such as dysphagia, gastrostomy tube dependency, xerostomia, soft tissue fibrosis, and secondary recurrence of radiation therapy due to higher overall survival rates.^106^, ^107^, ^108^ De-escalation trials are ongoing to demonstrate reduced treatment toxicity while preserving oncologic cure. Patients most likely to benefit from locoregional therapeutic de-escalation are those with low-risk disease, such as those with T1-T3, N0–N2b, and less than 10 pack-years of smoking.^106^ Cisplatin provides important prognostic findings and has a 90% 3-year survival rate.^109^^,^^110^ Diammineplatinum (II) dichloride, also known as cisplatin, is a DNA intercalator that targets cells that rapidly multiply. By attaching to the guanine residues, this intercalator develops DNA crosslinks that ultimately cause cell death. HPV patients are more likely to respond to platinum-based chemotherapy.^111^ Additional often-used chemotherapeutic medications include methotrexate; taxanes, including docetaxel and paclitaxel; methotrexate; and 5-fluorouracil.^110^^,^^112^^,^^113^ Chemotherapeutic medications are promising for treating patients with HNSCC, but research is ongoing for more effective drugs that target tumor cells directly. The targeted chemotherapy drugs such as cetuximab. This group is exploring the use of neoadjuvant chemotherapy followed by surgery for managing p16-positive treatment-naive oropharyngeal cancer. The treatment strategy involved de-escalation through transoral surgery and selective neck dissection, followed by systemic escalation using neoadjuvant chemotherapy (cisplatin/docetaxel).^114^

### Radiotherapy

The immune response to radiation is believed to be triggered by the increased presentation of antigens by the host immune cells.^115^ Chemotherapy and radiotherapy release inflammatory cytokines, such as tumor necrosis factor (TNF), interleukin (IL)-6, and IL-8, due to cell damage and inflammation caused by these treatments. In addition, damage-associated molecular patterns (DAMPs), such as high-mobility group box 1 (HMGB1), up-regulate phagocytosis-inducing signals, such as calreticulin, in dendritic cells.^116^ Radiation is a common treatment for HNSCC and is often incorporated into a multimodal therapeutic approach.^117^^,^^118^ Radiation therapy causes double-strand breaks in tumor cells, reducing cell viability and arresting more cells in their cycle, leading to cell death.^119^ Intensity-modulated radiation has been developed because of developments in radiotherapy.^120^^,^^121^ It reduces radiation exposure to non-cancerous cells while still delivering radiation to tumor tissues.^97^^,^^122^ It is a more efficient method for achieving a constant dose distribution, better tumor treatment, and careful healthy tissues.^121^ Radiation regimens aim to decrease dosage and therapy duration.^99^ Chemoradiation, performed after transoral robotic surgery as the initial surgical technique, can effectively manage both HPV-related and HPV-unrelated diseases in patients.^96^^,^^123^ These patients are susceptible to the adverse effects of both surgical and nonsurgical interventions.^96^^,^^124^ Most cervical and anal malignancies, which are believed to be HPV-related, also exhibit favorable radiosensitivity. The increased immunogenicity of HPV-related malignancies has been proposed as a potential explanation for the radiosensitivity observed in these diseases. Radiotherapy induces immunogenic cell death, enhances antigen presentation, causes inflammation, and triggers dendritic cells, which, in turn, stimulate cytotoxic T lymphocytes.^125^, ^126^, ^127^

### Treatment of cancer related to HPV

Understanding the treatment paradigm for HPV-positive oropharyngeal cancer can be enhanced by studying the past treatment methods for patients with oropharyngeal cancer. The transoral technique is commonly used to treat T1 and small T2 primary tumors of the upper oropharynx, including the tonsils, soft palate, and posterior pharyngeal wall.^128^ Clinicians are increasingly understanding the need for diverse treatment protocols for HPV and non-HPV patients because of their diverse etiologies.^109^^,^^124^^,^^129^^,^^130^ HPV expression has been linked to increased patient reactions to conventional chemotherapy, radiation, and radiochemotherapy. The 3-year survival rate for individuals with OPSCC is approximately 75%, which is comparable to that of individuals with non-HPV-related cancers.^131^, ^132^, ^133^, ^134^, ^135^ Research indicates that HPV-positive HNSCC can reduce recurrence by 50%, mortality risk by 40%, and metastasis incidence by 40% compared with HPV-negative HNSCC.^131^^,^^135^, ^136^, ^137^, ^138^ The longevity benefit observed in HPV patients may be due to molecular alterations resulting from virus-mediated activities rather than carcinogens or mutations in non-HPV patients.^139^^,^^140^ Prolonged use of therapeutic medicines can inhibit the E6 oncogene, allowing normal functioning of the TP53 gene.^111^^,^^141^ *In vitro* chemoradiotherapy improves survival and resistance to cancer cell lines compared with *in vivo* therapy, in which cells are surrounded by an immunological environment.^98^^,^^133^^,^^142^ Research shows that patients with HPV-infected tumors have higher T cell infiltration levels and higher percentages of HPV-specific cytotoxic CD8^+^ T cells than non-HPV tumor patients.^133^^,^^138^^,^^142^ The prognosis for OPSCC may differ based on HPV-related factors, as tumor colonies with intra-tumoral heterogeneity consist of subpopulations. Tumors with significant intra-tumoral heterogeneity often show suboptimal treatment response, recurrence, or metastasis, suggesting a higher receptiveness to treatment regimens.^98^ HPV-driven malignancies may respond better to treatment owing to their homogeneity and single-agent-induced population, but a suitable mono-dimensional therapeutic strategy for head and neck cancer remains unresolved.^124^ The increasing number of young patients may be significantly affected by long-term functional difficulties, despite the equal oncological effects of various therapeutic approaches. Clinicians often encounter patients who are likely to fully recover and outlive their disease, making them particularly susceptible to the late effects of cancer treatment.^98^^,^^131^^,^^132^^,^^143^^,^^144^ The concurrent administration of radiochemotherapy may lead to excessive therapy.^96^ Advanced techniques, such as adoptive T-cell therapy and chimeric antigen receptor-T immunotherapy, are being used to treat cancer, with researchers using CRISPR-based technology for checkpoint inhibition and chimeric antigen receptor-T immunotherapy to detect cancer cells.^145^

### Prophylactic vaccines

New HPV vaccines have the potential to prevent numerous infections and reduce the burden of HPV-associated diseases. Two vaccines have been developed using HPV L1 proteins, which self-assemble into virus-like particles. One vaccination uses virus-like particles from HPV-6, -11, -16, and -18, whereas the other uses virus-like particles from HPV-16 and -18.^146^ The rising prevalence of HNSCC caused by HPV necessitates the development of a vaccine to prevent oral HPV infection before malignant lesion development.^109^^,^^147^ Because of previous vaccinations, such as influenza and varicella, such prototypes should assist in the development of prophylactic measures against oral HPV infection.^109^ Preventive immunization against HPV in the cervix has been developed and is currently available to most people.^96^ Gardasil, the first licensed preventative vaccine, is a quadrivalent vaccine that protects against high-risk HPV-16 and -18, and low-risk HPV-6 and -11.^109^^,^^148^ The development of Cervarix was concentrated on a two-valent vaccine against HPV-16 and -18.^109^^,^^148^ The quadrivalent vaccine targets genital warts and common non-cancerous viral types, while both types of prophylaxis protect against dangerous HPV variations in cervical cancer.^98^^,^^109^ A study comparing Cervarix and Gardasil found that Cervarix was more effective in triggering an immune response against oncogenic HPV strains, excluding low-risk variations.^148^ Phase III trials have demonstrated the effectiveness and safety of these vaccines in preventing warts, lesions, and diseases caused by anogenital HPV.^96^^,^^109^ The efficacy of the HPV vaccine in preventing oral canal infections is supported by oral rinses collected during a trial that evaluated its efficacy in treating cervical infections.^149^, ^150^, ^151^, ^152^ Current preventive vaccinations effectively prevent HNSCC caused by HPV strains by protecting against the main HPV strains that cause OPSCC.^109^

### Therapeutic vaccines

**Figure 6:**
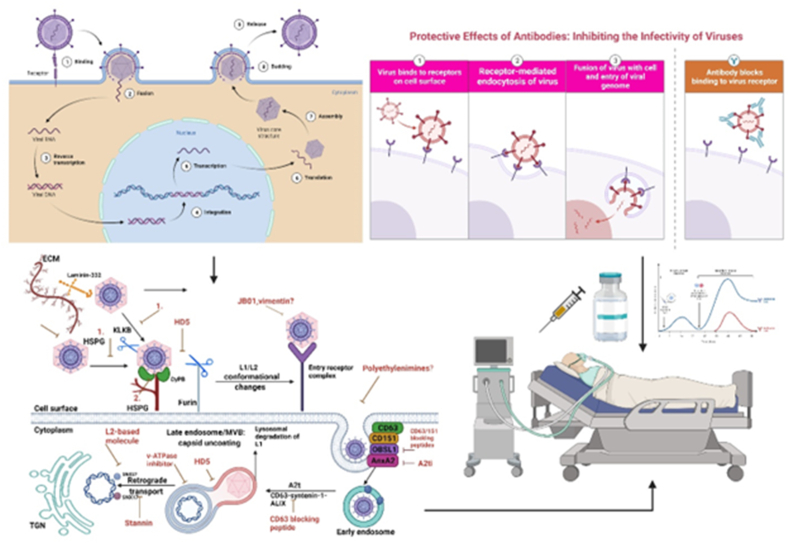
The virus's life cycle and a schematic representation of the early stages of HPV infection, showing the locations where different anti-HPV molecules are inhibited. In addition to curing patients, vaccines can prevent HPV.

HPV is linked to squamous cell carcinomas in various parts of the body, including the oropharynx, anus, rectum, penis, vagina, and vulva.^153^^,^^154^ HPV-induced diseases have been extensively studied and treated because of their significant role in oncogenesis.^155^ Therapeutic vaccinations activate the T cell-mediated immune system to kill cells infected with HPV, inhibiting cancer development, unlike preventative vaccines that cause antibody-mediated humoral reactions (Fig. 6).^148^^,^^156^ Immunocompromised patients face challenges owing to their poor immune system, making vaccinations best for immunocompetent individuals. HPV-16 E6 and E7 oncoproteins are key viral targets for the development of preventive vaccinations essential for transformation in HPV-associated cancers.^157^, ^158^, ^159^, ^160^ Viral E6 and E7 are entirely different from tumorigenic antigens, which are self-proteins. The expression of antigenic epitopes of these proteins helps boost the immune response.^157^^,^^161^^,^^162^ These viral proteins are ideal targets for treating HPV-induced malignancies, as they are expressed exclusively by infected cells.^156^^,^^157^ DNA vaccines are promising because of their stability, ease of preparation, and safety. However, they lacked immunogenicity. Novel approaches to increasing vaccine effectiveness include altering DNA-transfected dendritic cells, enhancing the connection between dendritic cells and T cells, and increasing antigen processing and presentation.^162^ Moreover, DNA vaccines introduce plasmid DNA into the host, promoting the transcription and immunological presentation of encoded HPV proteins in transfected cells.^156^^,^^162^ DNA vaccines may have limited immunogenicity because of their inability to amplify and transfer the DNA of transfected cells to the surrounding cells.^162^^,^^163^ Numerous clinical investigations have been conducted on therapeutic HPV DNA vaccination.^162^ A phase I trial is testing an HPV-16 E7 antigen-targeting DNA vaccine for individuals with advanced HPV-16 positivity.^105^^,^^162^ The activation of an antigen-specific T-cell response may lead to the eradication of infected cells.^159^ Peptide vaccines have poor immunogenicity despite being secure, simple to prepare, and safe to use.^159^^,^^162^ Adjuvants, such as Toll-like receptor (TLR) ligands, costimulatory molecules, cytokines, and chemokines, are used to compensate for poor immunogenicity.^162^^,^^163^ The constraint of using overlapping long peptides rich in antigen epitopes can be avoided using overlapping peptides.^163^ Protein and peptide vaccines have similarities because of their ability to bypass MHC restrictions owing to their diverse antigenic epitopes.^148^^,^^156^ Protein vaccinations containing MHC class II molecules typically trigger a humoral response rather than a cell-mediated response.^148^^,^^156^ A discovery of HPV vaccination was represented by TA-CIN as an HPV-16 E6, E7, and L2 fusion protein. It combines therapeutic and preventive vaccines.^158^^,^^163^ The vaccine production process involves removing underdeveloped dendritic cells by transfecting or pulsing antigen-expressing autologous dendritic cells, allowing them to develop, and then injecting them back into the patient.^162^^,^^163^ Antigens similar to live vectors, such as bacteria or viruses, can transfer substances from E6 and E7 oncoproteins to host antigen-presenting cells for enhanced presentation and cell-mediated reactions.^153^^,^^156^^,^^163^ A study developed a vector vaccine linking the HPV-16 E7 protein to calreticulin, which can cure 90% of early-stage cancers with a single intramuscular injection.^131^^,^^160^ The development of immunotherapeutic methods for HPV-associated intraepithelial neoplasia presents a promising opportunity to validate immune-based therapeutic approaches for epithelial malignancy. The accessibility of the lower genital area allows for the study of immunological treatment effects on the systemic circulation and target tissue.^164^ Figure 6 shows the HPV life cycle and a schematic representation of the early stages of infection, highlighting the sites at which various anti-HPV molecules exert their inhibitory effects. In addition to preventing HPV infection, vaccines may contribute to treatment.

### Currently available HPV vaccines

Approximately 70% of cervical cancer cases globally are due to HPV-16 and HPV-18 infections, with minor geographic variations.^165^ The remaining 30% are composed of approximately 11 distinct subtypes.^166^ HPV-16 and -18 are responsible for over 90% of non-cervical cancers linked to HPV, with HPV-16 being the majority. Cervical cancer and HPV-related diseases are among the numerous cancers with high mortality rates in many individuals annually. Although regular physicians are essential for HPV protection, vaccination is the most practical and cost-effective method. The L1 spontaneously produced virus-like particles are highly immunogenic and produce high titers of neutralizing antibodies to prevent HPV infection.^167^ Currently, available HPV vaccines are developed using a combination of various subtypes of L1-virus-like particles. The Merck vaccine uses yeast to express the viral L1 primary capsid protein, whereas the GSK vaccine uses insect cells for non-infectious subunit vaccine production.^42^ The vaccine administered by GSK is bivalent because it contains virus-like particles from HPV-16 and -18.^168^ The Merck vaccine is quadrivalent and contains virus-like particles from HPV-6, -11, -16, and -18. The global prevalence of cervical cancer has led to extensive international phase III clinical trials that focus on women. The Merck vaccine demonstrated significant defense against HPV-6 and HPV-11 genital warts, preventing recurrent incidental infection and premalignant genital disease associated with HPV16 and -18.^168^, ^169^, ^170^ The Merck vaccine appeared to protect against premalignant anal neoplasms and genital warts in smaller studies on male subjects. The Merck vaccine is approved for both genders aged 9–26, while the GSK vaccine is only approved for females aged 10–25 in the US and other countries. Clinical studies on the efficacy of HPV-associated immunization against oropharyngeal cancer are uncertain because of the lack of known premalignant lesions for screening.^171^ Therapeutic vaccinations can be used as a standalone therapy or in conjunction with surgery to enhance treatment outcomes and prevent recurrence. HPV therapeutic vaccines, including live vectors, proteins, nucleic acids, and whole cells, are expected to enhance the immune response. Most vaccines target antigenic targets E6 and E7 to trigger systemic cellular immunity and kill HPV-infected cells via cytotoxic T lymphocyte responses.^153^

### Clinical management and therapy

The incidence of global oropharyngeal cancer is increasing owing to HPV, a newly discovered causal agent of head and neck malignancies, particularly in the oropharynx. Patients with HPV-negative head and neck cancer generally have a worse prognosis than those with HPV-positive oropharyngeal cancer owing to various risk factors. New surgical techniques, such as transoral laser and robotic surgery, have revived the primary surgical treatment for HPV-positive individuals. Clinical trials are underway to develop optimal treatment plans for a growing population of HPV-positive oropharyngeal cancer patients. Identifying HPV-positive cancer patients who are at risk for survival and recurrence is crucial for customizing treatment plans and preventing undertreatment.^128^ The clinical manifestations of HPV infection are managed rather than the virus itself. Anogenital warts can be treated with both prescription and over-the-counter medications, depending on the specific disease. Patients with extensive genital, rectal, or cervical warts, or those who continue to have anogenital warts after regular treatment, should consult a doctor. Doctor-prescribed medications for genital wart treatment include suggested dosage and treatment course, and patients could use catechins (15%), podofilox (0.5% solution or gel), imiquimod (3.75%), and ointment (5 drugs).^172^ Follow-up appointments to assess medication use and treatment outcomes are recommended after a few weeks of treatment. Medical professionals recommend surgical removal, trichloroacetic acid, and cryotherapy. Despite limited information and evidence on their harmful effects, numerous provider-administered therapies are available. Recent care guidelines recommend follow-up care for cervical squamous intraepithelial lesions that can be removed using techniques such as tangential scissor excision and electrosurgery.^173^ High-grade cervical intraepithelial neoplasia (CIN-2 or CIM-3) can be treated using lasers, cryotherapy, conization, or loop electrosurgical excision, depending on the patient's condition. The two most popular methods for treating adenocarcinoma *in situ* are hysterectomy and conisation. Cervical cancer treatment includes surgery, radiation, chemotherapy, and other methods depending on the patient's age, tumor size, stage, and childbirth preferences. However, effective therapies for high-grade anal intraepithelial neoplasia are limited. The patient was receiving treatments such as 5-fluorouracil and intra-anal imiquimod, while the provider used surgical treatment, trichloroacetic acid, electrocautery, and infrared coagulation. According to retrospective studies,^174^, ^175^, ^176^ the per-lesion cure rates following initial therapy range from 63% to 85%, depending on therapeutic strategy. There is a lack of knowledge regarding the effect of high-grade anal intraepithelial neoplasia treatment on the incidence of anal cancer, cancer-related morbidity, and death. Treatment options for anal cancer may include local excision alone, excision combined with radiation and chemotherapy, or more complex surgical procedures, such as abdominoperineal resection.^177^ These options vary depending on tumor stage and location. Cold-knife conization for stage IA1 is the first fertility-sparing procedure, with successful cure rates and subsequent pregnancies.^178^ Stage IA1 disease has a minimal risk of pelvic lymph node involvement (1%), whereas stage IA2 has an 8% risk and 33% lymph vascular space invasion, making cautious strategies challenging.^179^ The first preventative HPV vaccine was primarily distributed among wealthier nations. The first two successful vaccinations resulted in the vaccination of hundreds of millions of women and a smaller number of males. Immunity is gaining traction in high-vaccination countries, such as Australia, the UK, and other regions, with the clinical effects of vaccination programs already evident. Vaccines have a great safety record and are more effective than initially believed. In 2015, the second-generation HPV virus-like particle vaccine Gardasil9 was approved and is currently on the horizon. The primary concerns surrounding HPV vaccination include the duration of protection, cross-protection, response duration, and protection against oropharyngeal HPV infections.^180^ A study found benefits of second-generation HPV vaccines, which are expected to extend protection to five more oncogenic types. The results showed that, assuming full adoption of the second-generation vaccine, lifetime cancer risk reductions in Kenya and Uganda were 86.3% and 91.8%, respectively. Second-generation HPV vaccines may improve cervical cancer prevention by protecting against oncogenic viral strains. The effectiveness of vaccines may be affected by co-infection with multiple HPV strains and unidentified strains, but cross-protective effects may mitigate this effect.^181^

### Control and prevention

Currently, phase II and III trials have investigated and developed HPV preventive vaccinations.^182^^,^^183^ The US has approved two HPV vaccinations: Gardasil, a quadrivalent vaccine for women and men aged 9–26 years, and Cervarix, a bivalent vaccine for females aged 9–25 years. Both vaccines are non-contagious, antibiotic-free, and do not contain thimerosal or mercury.^184^^,^^185^ Boys aged 11–12 should regularly receive the quadrivalent vaccine, whereas those aged 13–21 who have not received one or more doses are advised to be vaccinated.^186^ Men who have sex with men are required to receive a shot yearly until the age of 26.^186^ The vaccine should be administered to individuals with HIV of both sexes and other immunocompromised populations up to the age of 26 years. Both vaccines have demonstrated remarkable success in the prevention of specific cervical precancers. Quadrivalent and bivalent HPV vaccines have demonstrated over 93% effectiveness in preventing high-grade cervical intraepithelial neoplasia (CIN-2 or CIN-3) and adenocarcinoma *in situ* after clinical testing.^187^^,^^188^ The quadrivalent HPV vaccine demonstrated exceptional efficiency in preventing anogenital warts, anal precancers, and vaginal/vulvar precancers.^189^^,^^190^ Participants with HPV seropositive and polymerase chain reaction-negative genotypes had higher anti-HPV-6, -11, -16, and -18 geometric mean titers than those aged 16 to 23.^17^ Trials show strong antibody titers to various vaccines up to the age of 8 years, with ongoing research observing immunization recipients' cohorts to track effectiveness and immunogenicity. HPV safety profile vaccinations have been shown to be satisfactory during pre- and post-licensure safety investigations.^191^^,^^192^ Prelicensure safety evaluations have found that erythema, edema, and pain at the injection site are the most common local side effects of vaccines. More than 56 million quadrivalent HPV vaccination doses have been administered in the US.^192^ Prophylactic immunization is most effective when administered in advance or before a person has their first sexual contact. Immunization is not effective in treating diseases that have already manifested.^193^ Proper and frequent use of condoms can decrease the risk of HPV and related diseases, such as genital warts and cervical cancer.^194^ Preventing genital HPV infection involves avoiding sexual activity and contact with others, selecting sexually active individuals, and limiting sexual partners. Counseling and understanding cervical disease prevalence are crucial for effective vaccination and cervical cancer screening.^182^^,^^183^

## Conclusion and future perspectives

The widespread use of preventive vaccines has significantly improved the prevention and treatment of HPV-associated cervical cancer linked to HPV. The outline of HPV vaccination, including bivalent, quadrivalent, and nonavalent formulations, has significantly reduced the prevalence of high-risk HPV infections and related precancerous lesions. Furthermore, the global potential to reduce the incidence of cervical cancer and other HPV-related malignancies has grown with the expansion of vaccination programs to include both sexes and diverse age groups. Advancements in immunotherapy, gene editing, and therapeutic vaccination are promising for the treatment of HPV infection and cancer. Investigation of therapeutic techniques, such as CRISPR/Cas9, siRNA-based methods, and immune checkpoint inhibitors, is underway to remove HPV-infected cells and stimulate host immune responses. Advancements in health care are shifting from prevention-focused paradigms to integrated strategies that combine therapeutic and preventive measures. Future research should focus on developing vaccines that are comprehensive, cost-effective, thermostable, and that cover a wider range of HPV genotypes. Additionally, the integration of personalized immunotherapies and therapeutic vaccinations into clinical practice has the potential to revolutionize the treatment of HPV-associated cancers. Furthermore, the integration of targeted treatments with molecular diagnostics may potentially lead to precision medicine in HPV oncology. The global elimination of HPV-related diseases necessitates multifaceted international efforts involving researchers, policymakers, medical professionals, and public health activists.

## CRediT authorship contribution statement

**Md Rezaul Islam:** Conceptualization, Writing – original draft. **Abdur Rauf:** Writing – review & editing, Writing – original draft, Supervision, Methodology, Investigation, Conceptualization. **Most Nazmin Aktar:** Data curation, Formal analysis, Methodology. **Md Naeem Hossain Fakir:** Methodology, Writing – review & editing. **Sadiya Islam Trisha:** Methodology, Writing – original draft. **Asraful Islam Asif:** Validation, Writing – original draft. **Md Harun Or Rashid:** Data curation, Resources. **Md Ibrahim Khalil Al-Imran:** Formal analysis, Methodology. **Gazi Kaifeara Thufa:** Resources, Software. **Farhana Prodhan Emu:** Project administration, Resources, Visualization. **Hassan A. Hemeg:** Validation, Writing – review & editing. **Hanan A. Ogaly:** Validation, Writing – review & editing. **Rekha Thiruvengadam:** Writing – review & editing, Software, Resources, Methodology. **Seung-Hyun Kim:** Supervision, Writing – review & editing. **Muthu Thiruvengadam:** Writing – review & editing, Writing – original draft, Validation, Supervision, Investigation, Conceptualization.

## Conflict of interests

The authors declare that they have no known competing financial interests or personal relationships that could influence the work reported in this study.
